# Sellar Lesions: Novel Aspects in Diagnosis and Management

**DOI:** 10.3390/cancers18061029

**Published:** 2026-03-23

**Authors:** Georgios Kostopoulos, Evangelia S. Makri, Efstathios Divaris, Zoe A. Efstathiadou

**Affiliations:** Department of Endocrinology, “Ippokration” General Hospital of Thessaloniki, 54642 Thessaloniki, Greece; gnkostop@auth.gr (G.K.); msevange@auth.gr (E.S.M.); edivari@auth.gr (E.D.)

**Keywords:** pituitary neuroendocrine tumor (PitNET), pituitary adenoma, sellar lesions, transcription factors, sella turcica, classification, molecular markers, treatment

## Abstract

In this comprehensive review, we explore the evolving landscape of research and clinical practices in sellar lesions, emphasizing recent advancements in histopathology and molecular biology. Distinct lesions can arise from the sellar area, predominantly comprising different tumor types, inflammatory conditions, or systemic conditions. The recent CNS5 World Health Organization classification integrates genetic modifications into histopathological characteristics, enhancing the ability to predict the biological behavior and malignant potential of these lesions. Furthermore, the molecular alterations discovered in these tumors may act as valuable diagnostic and prognostic indicators, facilitating a tailored approach, especially for those demonstrating aggressive characteristics resistant to conventional treatments. The scope of the present review is to provide a comprehensive insight into the current understanding of sellar lesions with regard to emerging prognostic factors, like molecular alterations, to advances in clinical strategies, and to identification of potential new therapeutic targets within the oncology field.

## Simple Summary

Sellar lesions include a wide range of tumors and non-tumor conditions that can be difficult to diagnose and manage. Recent advances in molecular biology have increased our knowledge regarding the mechanisms underpinning pituitary tumorigenesis and proliferation, leading to the development of novel classification systems and diagnostic and treatment modalities. Integration of research data into clinical practice may lead to more accurate diagnosis, better knowledge of the biological behavior of sellar lesions and personalized treatment.

## 1. Introduction

Sellar lesions constitute a diverse group of neoplastic and non-neoplastic lesions arising within one of the most anatomically complex regions of the skull base. Pituitary adenomas account for more than 80–90% of all sellar masses, followed by other non-adenomatous lesions, such as Rathke’s cleft cyst (RCC), craniopharyngioma, meningioma, and a range of rare neoplasms [1,2,3]. Clinically, sellar lesions present with diverse manifestations that reflect their biological behavior and anatomical relationships [1,4]. The spectrum of clinical manifestations ranges from an asymptomatic, incidentally identified lesion in the anterior or posterior pituitary lobe to endocrine dysfunction and mass effects, such as headache and visual defects. Although the majority of them constitute benign tumors, they are associated with significant morbidity and mortality [1,4].

Timely and accurate recognition of sellar lesions is crucial for guiding appropriate and effective management. The diagnosis of pituitary tumors primarily relies on a combination of clinical examination, endocrine evaluation, and neuroimaging [1,2,3,4]. However, diagnostic accuracy may be limited by the substantial overlap in clinical manifestations and radiological features among different lesions. On such occasions, histopathological confirmation remains the gold standard for definitive lesion classification [5,6].

Emerging knowledge regarding the molecular mechanisms implicated in the pathogenesis of pituitary lesions has led to significant advancements in diagnosis, prognosis and therapeutic approaches. Novel, evidence-based classification systems incorporating clinical (PANOMEN-3) and histopathological (WHO CNS5) criteria have been developed to diminish diagnostic uncertainty and improve patients’ outcomes [7,8,9]. Therefore, our aim was to provide a state-of-the-art, comprehensive overview of current evidence regarding the diagnosis and management of sellar lesions.

## 2. Classification and Prevalence

The term sellar lesions encompasses a broad spectrum of neoplastic and non-neoplastic conditions, each distinguished by unique clinical, radiological, and pathological characteristics (Table 1). Pituitary neuroendocrine tumors (PitNETs) (formerly pituitary adenomas), the most prevalent sellar lesions, are typically benign neoplasms originating from the anterior pituitary gland [10]. PitNETs are further categorized based on hormonal activity and cell type, including prolactinomas, growth hormone (GH)-secreting adenomas, adrenocorticotropic hormone (ACTH)-secreting adenomas, and non-functioning adenomas [9]. RCCs, benign fluid-filled cysts derived from remnants of Rathke’s pouch, can enlarge and exert mass effect on the pituitary gland or optic chiasm. Meningiomas, tumors arising from the meninges, can also occur in the sellar region, frequently presenting with visual disturbances and hormonal dysfunction [11]. Craniopharyngiomas, slow-growing and histologically benign tumors arising from epithelial remnants of the Rathke’s pouch, are associated with significant neurological, endocrine, and visual comorbidities [12]. Metastatic tumors to the pituitary gland, while relatively uncommon, must be considered in patients with a history of systemic malignancy. Less prevalent sellar lesions include pituitary abscesses, granulomatous diseases, and vascular lesions. Solitary fibrous tumors, though rare, have been reported in the sellar and suprasellar regions, presenting diagnostic and operative challenges [13]. Several subtypes of hypophysitis can present as sellar masses and closely mimic PitNETs both clinically and radiologically. Lymphocytic hypophysitis, the most common form, typically affects young women and may present with symmetrical pituitary enlargement and multiple pituitary hormone deficiencies. Granulomatous hypophysitis, either idiopathic or secondary to systemic diseases such as sarcoidosis or tuberculosis, often shows imaging features indistinguishable from PitNETs. Xanthomatous hypophysitis is rare and may appear as a cystic sellar lesion, whereas necrotizing hypophysitis, although extremely uncommon, can present as an acute pituitary mass. IgG4-related hypophysitis, more frequently seen in older patients, and immune checkpoint inhibitor–related hypophysitis further expand the differential diagnosis of PitNETs. Recognition of these inflammatory entities is essential to avoid misdiagnosis and inappropriate surgical intervention [14,15]. Accurate diagnosis of sellar lesions necessitates a multidisciplinary approach, integrating clinical evaluation, radiological imaging, and histopathological analysis. Distinguishing between various etiologies can be challenging due to overlapping clinical, endocrinological, and radiographic presentations, often mimicking PitNETs [16].

### 2.1. WHO CNS5 Classification-Updates (Diagnostic Criteria)

#### Updates (Diagnostic Criteria)

The 5th edition of the World Health Organization Classification of Central Nervous System Tumors introduces significant updates to the classification and grading of pituitary tumors, reflecting advancements in molecular diagnostics and clinical understanding (Table 1). The updated classification emphasizes the importance of integrated diagnosis, incorporating histopathological features, immunohistochemical markers, and genetic alterations to refine tumor classification and predict clinical behavior [9].

The 2021 WHO CNS5 classification highlights several key points regarding pituitary tumors: (a) Cell lineage and transcription factor emphasis: The categorization of pituitary adenomas is now more closely aligned with their cell lineage and the expression of specific transcription factors, influencing the nomenclature of adenoma subtypes; (b) Integrated diagnostic approach: The WHO CNS5 advocates for an integrated diagnostic process that combines histopathological findings, immunohistochemical analysis, and genetic alterations; (c) Atypical pituitary adenoma criteria: The definition of atypical pituitary adenoma has been updated to incorporate factors such as mitotic activity, p53 immunoreactivity, and the Ki-67 labeling index.

**Figure 1:**
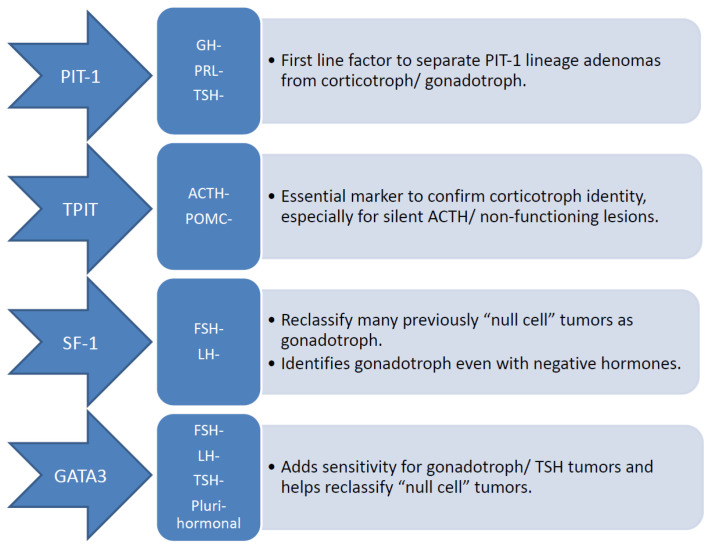
Pituitary adenomas according to transcription factors. Pituitary transcription factors are routinely employed in pathology, where PIT-1 identifies somatotroph, lactotroph, and thyrotroph tumors; TPIT marks corticotrophs; and SF-1 indicates gonadotroph tumors. GATA-3 serves as an adjunct nuclear marker, enhancing the detection of gonadotroph and certain TSH-lineage tumors. The application of nuclear immunohistochemistry for these transcription factors improves lineage assignment and consequently reduces the number of tumors categorized as true null-cell PitNETs. ACTH, Adrenocorticotropic Hormone; FSH, Follicle-Stimulating Hormone; GH, Growth Hormone; LH, Luteinizing Hormone; PitNET, Pituitary Neuroendocrine Tumor; POMC, Proopiomelanocortin; PRL, Prolactin; TSH, Thyroid-Stimulating Hormone.

Regarding pituitary adenomas, WHO CNS5 adheres to the framework established in the fourth edition of the WHO classification of endocrine tumors, categorizing them according to adenohypophyseal cell lineage based on combined immunohistochemical expression of pituitary hormones and lineage-specific transcription factors. The classification also incorporates the term PitNET, introduced by the WHO endocrine tumor group, which remains a subject of ongoing discussion for future editions [17]. In routine neuropathological practice, pituitary transcription factor expression is essential in tumor classification and lineage determination. PIT-1 is used to identify tumors of somatotroph, lactotroph, and thyrotroph differentiation, whereas TPIT serves as a marker of corticotroph lineage and SF-1 denotes gonadotroph tumors. In addition, GATA-3 functions as a complementary nuclear marker, facilitating the recognition of gonadotroph tumors and a subset of neoplasms within the thyrotroph lineage [9] (Figure 1).

In earlier classifications, adamantinomatous and papillary craniopharyngiomas were regarded as variants of a single tumor entity. In the current WHO CNS5 classification, however, they are recognized as distinct tumor types, reflecting differences in clinical presentation, patient demographics, radiologic appearance, histopathology, molecular alterations, and DNA methylation profiles [18].

By contrast, pituicytoma, granular cell tumor, and spindle cell oncocytoma are grouped together within a single category of related tumors. These entities may represent morphological variants along a shared tumor spectrum; however, differences in demographic characteristics and clinical behavior may justify their continued classification as separate tumor types [18].

Finally, pituitary blastoma, a rare embryonal tumor of infancy, has been newly recognized as a distinct tumor entity in the WHO CNS5 classification [9].

### 2.2. PANOMEN-3 Classification System

Application of the WHO CNS5 system is only feasible when a pituitary tumor is surgically resected. However, observational data highlight that a substantial proportion of cases (almost 50%) are not surgically treated. To address this gap, Ho et al. developed a prognostic model incorporating clinical, biochemical and histopathological features for risk stratification [7]. Findings from validation studies suggest that the PANOMEN-3 prognostic model showed promising performance in predicting tumor behavior in both unresected and resected pituitary tumors [8,19].

This classification system evaluates multiple factors that may influence morbidity, including the patient’s clinical phenotype, genetic syndromes, hormone secretion status, presence of hypopituitarism, tumor size, mass effect, and local invasion, in order to grade pituitary tumors before surgery. Postoperatively, it also considers residual tumor and histopathological findings. Each factor is assigned a score, and all added up contribute to an overall corrected score ranging from 0 to 3. These corrected scores provide a standardized measure of disease severity, which is then used to classify tumors into four grades: Grade 0: corrected score = 0; Grade 1: corrected score > 0 and < 0.3; Grade 2: corrected score ≥ 0.3 and ≤0.6; and Grade 3: corrected score > 0.6. This grading system allows clinicians to stratify patients from minimal disease severity to severe disease and from low to high morbidity/mortality risk. Hence, PANOMEN-3 may have the potential to serve as a valuable decision-support tool for both endocrinologists and neurosurgeons [8,19].

### 2.3. Prevalence

The spectrum of sellar lesions displays notable variability in prevalence. PitNETs, with a prevalence of 77 to 94 cases per 100,000 individuals, represent the predominant type of sellar lesions, constituting approximately 90% of all sellar disorders and, in general, constituting 10–15% of all intracranial tumors [10,20,21]. Prolactinomas are the most frequently observed, accounting for approximately 40% of all PitNETs [20]. Non-functioning pituitary adenomas (NFPAs), characterized by the absence of hormone secretion, constitute 15–30% of PitNETs [22]. GH-secreting adenomas, responsible for acromegaly, constitute approximately 20% [23]. ACTH-secreting adenomas, which cause Cushing’s disease, are less prevalent, representing about 10–15% of PitNETs [24].

Additionally, non-pituitary lesions such as RCCs, the second most prevalent, are relatively frequent incidental findings at autopsy, with reported prevalence rates of up to 30%, and the craniopharyngiomas are frequently encountered [16,25,26]. Other non-neoplastic entities, such as pituitary abscesses and granulomatous diseases, are relatively uncommon but should be considered in the differential diagnosis of sellar lesions. Pituitary abscess is considered an uncommon yet potentially fatal disorder, accounting for approximately 0.2–1.1% of surgically treated pituitary lesions [27].

Among benign tumors, meningiomas are most frequent, following craniopharyngiomas [2].

Pituitary carcinomas, although exceedingly rare, are histopathologically indistinguishable from pituitary adenomas and are defined by the presence of distant metastases [28]. These tumors account for less than 1% of all pituitary neoplasms and typically exhibit aggressive behavior.

### 2.4. Clinical Manifestations

Endocrine abnormalities resulting from sellar lesions can manifest in various ways, depending on the specific hormones affected. Hypersecretion of pituitary hormones can lead to a variety of clinical syndromes, including acromegaly (excess GH), Cushing’s disease (excess ACTH), and hyperprolactinemia (excess prolactin) [29]. Mass effect from pituitary lesions can compress the optic chiasm, leading to visual field defects, or impinge on the normal pituitary gland, resulting in hypopituitarism; the mass effect can also cause headaches, cranial nerve palsies, and, in rare cases, hydrocephalus. Pituitary lesions can disrupt the hypothalamic-pituitary axis, leading to hormonal imbalances and associated symptoms; giant pituitary adenomas, defined as those with a diameter greater than 4 cm, can cause headaches, dizziness, vision loss, and endocrine abnormalities [30].

### 2.5. Differential Diagnosis of Sellar Lesions

The differential diagnosis of sellar lesions encompasses a broad spectrum of neoplastic and non-neoplastic conditions, each distinguished by unique clinical, radiological, and pathological characteristics. PitNETs, the most prevalent sellar lesions, are typically benign neoplasms originating from the anterior pituitary gland [10]. PitNETs are further categorized based on hormonal activity and cell type, including prolactinomas, GH-secreting adenomas, ACTH-secreting adenomas, and NFPAs [9]. RCCs, benign fluid-filled cysts derived from remnants of Rathke’s pouch, can enlarge and exert mass effect on the pituitary gland or optic chiasm. Meningiomas, tumors arising from the meninges, can also occur in the sellar region, frequently presenting with visual disturbances and hormonal dysfunction [11]. Craniopharyngiomas, slow-growing and histologically benign tumors situated in the sellar-suprasellar region, are associated with significant neurological, endocrine, and visual comorbidities [12]. Metastatic tumors to the pituitary gland, while relatively uncommon, must be considered in patients with a history of systemic malignancy. Less prevalent sellar lesions include pituitary abscesses, granulomatous diseases, and vascular lesions. Solitary fibrous tumors, though rare, have been reported in the sellar and suprasellar regions, presenting diagnostic and operative challenges [13]. Several subtypes of hypophysitis can present as sellar masses and closely mimic PitNETs both clinically and radiologically. Lymphocytic hypophysitis, the most common form, typically affects young women and may present with symmetrical pituitary enlargement and multiple pituitary hormone deficiencies. Granulomatous hypophysitis, either idiopathic or secondary to systemic diseases such as sarcoidosis or tuberculosis, often shows imaging features indistinguishable from PitNETs. Xanthomatous hypophysitis is rare and may appear as a cystic sellar lesion, whereas necrotizing hypophysitis, although extremely uncommon, can present as an acute pituitary mass. IgG4-related hypophysitis, more frequently seen in older patients, and immune checkpoint inhibitor–related hypophysitis further expand the differential diagnosis of PitNETs. Recognition of these inflammatory entities is essential to avoid misdiagnosis and inappropriate surgical intervention [14,15]. Accurate diagnosis of sellar lesions necessitates a multidisciplinary approach, integrating clinical evaluation, radiological imaging, and histopathological analysis. Distinguishing between various etiologies can be challenging due to overlapping clinical, endocrinological, and radiographic presentations, often mimicking PitNETs [16].

In addition to imaging findings, the acuity of symptom onset and the clinical context at presentation may provide important diagnostic clues in the preoperative presentation of sellar lesions. Certain entities demonstrate characteristic clinical patterns. For example, sellar atypical teratoid/rhabdoid tumors frequently present with acute onset headache and rapidly progressive visual disturbances, reflecting their aggressive nature, whereas hypophysitis often occurs in specific clinical settings, such as the postpartum period or in association with autoimmune disease [31]. Therefore, integrating imaging characteristics with the clinical context and symptom onset may aid in raising early diagnostic suspicion and leading to appropriate management [32]. The clinical differentiation between sellar lesions is crucial due to the divergent therapeutic strategies for pituitary and non-pituitary lesions [16]. For instance, pituitary metastases, although infrequent, can present similarly to PitNETs and are often misdiagnosed because of comparable clinical manifestations and non-specific magnetic resonance imaging (MRI) findings [33,34]. However, indicators such as arginine-vasopressin deficiency, ophthalmoplegia, and variable anterior pituitary hormone deficiencies, particularly in patients with a known history of systemic malignancy, should prompt consideration of metastatic involvement of the pituitary gland [35]. These metastatic tumors are predominantly observed in elderly patients with widespread malignant disease, with breast and lung cancers being the most common primary origins [36,37]. Other primary tumor sites that may metastasize to the pituitary include renal cell carcinoma, gastrointestinal malignancies, and melanoma [38]. In certain situations, pituitary metastasis might represent the initial clinical presentation of an occult malignancy, necessitating a comprehensive investigation to identify the primary tumor [35].

In addition to primary pituitary tumors, the pituitary gland can be affected by inflammatory or infiltrative conditions that mimic neoplastic lesions and complicate diagnosis. Examples include sarcoidosis, Langerhans cell histiocytosis, and lymphocytic hypophysitis, which can manifest with symptoms ranging from diabetes insipidus to varying degrees of anterior pituitary insufficiency, requiring careful diagnostic consideration [10]. Moreover, although uncommon, certain genetic syndromes such as DICER1 syndrome can predispose individuals to pituitary blastomas, presenting with a combination of symptoms including ophthalmoplegia and signs of elevated intracranial pressure [10].

The differential diagnosis of sellar masses is summarized in Table 1, highlighting that while PitNETs are the most frequent cause of intrasellar masses, other etiologies warrant consideration [16]. Conditions such as craniopharyngiomas, meningiomas, RCCs, and metastatic lesions require careful evaluation alongside primary PitNETs [16,39]. Accurate differentiation is crucial due to the diverging management strategies, particularly for uncommon presentations like pituitary metastases, which can exhibit clinical and radiological similarities to adenomas [40]. Consequently, a heightened index of suspicion is imperative, especially in patients with a history of malignancy, as pituitary metastases may manifest with visual field defects, cranial nerve palsies, and pituitary dysfunction [41,42].

## 3. Advancements in Diagnostic Evaluations

The diagnosis of sellar lesions remains a complex and evolving field, reflecting the anatomical intricacy of the sellar region and the diverse pathologies that can arise within it.

### 3.1. Update MRI Protocols and Radiomics

MRI remains the cornerstone of initial imaging of sellar lesions, offering superior soft-tissue contrast and anatomical detail. Standard imaging of the sellar region consists of T1-weighted fast spin echo (FSE T1), before and after gadolinium administration, and T2-weighted images in coronal and sagittal planes with 2 mm slice intervals using a 1.5 T–3 T magnetic field. These sequences evaluate sella homogeneity and isointensity relative to gray matter, as well as signal intensity of the surrounding structures such as the optic chiasm and cavernous sinus. For example, PitNETs are hypointense in T1-weighted images, while signal intensity on T2-weighted images may vary. Additionally, cystic lesions are usually hypointense on T1- and hyperintense on T2-weighted images, respectively. Hyperintensity on T1-weighted images typically reflects high protein (RCC), blood (apoplexy) lipid content (lipoma) or calcifications (craniopharyngiomas), while T2 hypointensity is a hallmark of lymphocytic hypophysitis. Gadolinium enhancement pattern is often helpful in the differential diagnosis, as normal pituitary and infundibulum enhance homogeneously, whereas PitNets demonstrate delayed enhancement (hypointense in the early phase) and RCCs have no enhancement. Other signs to consider during evaluation include sellar enlargement, stalk displacement or thickening, absence of the posterior lobe bright-spot in T1-weighted scans, displacement of the sellar diaphragm, suprasellar extension and cavernous and sphenoid sinus extension [5].

Ultra-high field MRI, (7 T or higher), has shown greater sensitivity in detecting microadenomas in comparison with conventional 1.5 T or 3 T MRIs, but evidence is currently limited due to the small number of published cases [43,44]. Notably, higher resolution imaging comes inevitably at the cost of lower specificity, due to a higher signal-to-noise ratio and a greater number of incidentally discovered lesions.

Applications of alternative MRI sequences, such as dynamic enhanced MRI (dMRI) (T1-weighted images obtained before and after gadolinium injection at short sequential intervals), volumetric gradient echo (3D-GRE MRI) (high-resolution anatomical volumes in short scan intervals), diffusion weighted imaging (DWI) (measurement of water motion reflecting cellularity and cyst content) and fluid-attenuated inversion recovery (FLAIR) (cerebrospinal fluid suppression signal) may enhance the sensitivity in sellar masses detection [5]. MR elastography (evaluation of tumor consistency) may also predict treatment response in patients with macroadenoma before surgery [45].

MRI may reliably differentiate pituitary adenomas from other lesions, such as craniopharyngiomas, meningiomas, germ-cell tumors, cysts, metastases and pituitary hyperplasia based on signal intensity and contrast enhancement, as previously mentioned. In many cases, though, definitive diagnosis can be challenging, as many lesions may present with overlapping radiological features. Hence, integration of radiomics and machine learning (ML) models in clinical practice may help solve diagnostic dilemmas [6].

Radiomics enables the extraction of quantitative features from medical images, such as shape, texture and histogram features, using artificial intelligence (AI). These features correlate with tumor consistency, invasiveness and functional status, predicting tumor functionality and behavior [46,47]. Emerging data from retrospective studies highlight that radiomics may have high accuracy in differentiating between common sellar lesions with a sensitivity of 87.9% to 100% and specificity of 93.9% to 100% [46,48,49,50]. In fact, texture analysis was associated with lesion type, PitNet subtype, PitNet granulation pattern and consistency. For instance, smoothness and uniformity were more common in cases with NFPA compared to functioning PitNETs [47]. Furthermore, radiomics may reliably predict tumor recurrence in NFPAs with an accuracy greater than 85% and identify cavernous sinus invasion [46,51,52]. For example, low sphericity was associated with tumor aggressiveness, while texture analysis may be an indicator of Ki67 proliferation index [5,53]. Also, higher mean pixel intensity in NFPA was linked to lower recurrence rates [54]. Other applications of radiomics may include the prediction of treatment response with dopamine agonists (DAs) in prolactinomas and somatostatin receptor ligands (SRLs) in somatotroph adenomas. Park et al. developed and validated a radiomics ensemble classifier incorporating 30 radiomics features. The ensemble classifier outperformed conventional MRI features (T2 signal intensity and cystic/hemorrhagic change) in predicting treatment resistance to DA in patients with prolactinomas [55]. Moreover, T2 MRI hypointensity, which has been correlated with tumor SSTR2 expression and granulation pattern, is a predictor of good response to first-generation SRLs in patients with acromegaly [56]. Accordingly, maximum pixel intensity on T1-weighted images of patients with GH-secreting adenomas (another surrogate marker for granulation pattern) was also an indicator of biochemical response to SRLs [57].

In conclusion, although current evidence is promising, the role of radiomics remains investigational. Widespread clinical adoption will depend on overcoming challenges in reproducibility, interpretability, and validation across diverse patient populations.

### 3.2. Inferior Petrosal Sinus Sampling (IPSS)

Despite major advances in MRI techniques, small corticotroph tumors may remain undetectable or indistinguishable from incidental pituitary lesions. In such cases, additional diagnostic procedures may be required to confirm the source of ACTH secretion. Inferior petrosal sinus sampling (IPSS) remains the gold standard for distinguishing pituitary Cushing disease (CD) from ectopic ACTH secretion. Recent advances have focused on enhancing diagnostic accuracy while improving procedural efficiency and cost-effectiveness. Corticotropin-releasing hormone (CRH) was historically used as the stimulatory agent; however, because of its limited global availability, desmopressin (DDAVP) has emerged as a widely used alternative [58]. A recent meta-analysis demonstrated similar diagnostic performance between DDAVP and CRH for the diagnosis of ACTH-dependent CS (pooled sensitivity 96% vs. 98%, pooled specificity 100% vs. 100%) [59]. In addition, the use of prolactin-adjusted ACTH ratios as markers of adequate venous efflux has been proposed to reduce false-negative results and improve the interpretation of IPSS findings [60]. Simplified sampling strategies have also been explored; emerging data suggest that two-point sampling protocols with DDAVP stimulation may achieve diagnostic accuracy comparable to conventional multi-time-point protocols while reducing procedural complexity and cost [61]. In contrast, the utility of IPSS for lateralizing corticotroph adenomas remains limited, as its accuracy is modest and it does not reliably predict postoperative remission [62].

### 3.3. Functional Imaging

Functional imaging of sellar masses has evolved into a vital adjunct improving diagnostic precision, especially in cases with equivocal findings, negative cross-sectional imaging and postoperative evaluation. Over the past decades, there has been a growing body of evidence highlighting the potential role of functional imaging in the diagnosis of pituitary and, particularly, corticotroph adenomas.

Several radiotracers highlight distinct physiological features of pituitary lesions. Dopamine-2 receptor imaging (performed with radiolabeled D2R antagonists such as ^123^I-iodobenzamide) can identify prolactinomas, though its clinical impact is limited as most prolactinomas are managed medically without the need for imaging confirmation [63]. Somatostatin (SST) receptor–based imaging (e.g., indium-pentetreotide or newer ^68^Ga-labeled SST analogs) is hindered by heterogeneous SST expression among pituitary adenomas and significant uptake in the normal gland, reducing specificity. However, in select situations, such as suspected ectopic ACTH secretion, SST imaging may provide useful complementary information [64,65].

Among currently available modalities, ^11^C-methionine PET/CT or PET/MR has emerged as the most consistently informative functional technique for pituitary lesions [66]. By exploiting increased amino-acid uptake and protein synthesis in adenomas, Met-PET can help localize microadenomas in Cushing’s disease, clarify equivocal prolactinoma masses, and differentiate residual tumor from postoperative change [67]. Its value becomes especially pronounced in patients with persistent or recurrent disease after surgery or radiotherapy, when identifying the exact site of residual tumor is essential for planning repeat transsphenoidal exploration or stereotactic radiosurgery [67]. Functional imaging has also shown benefit in challenging cases of acromegaly, particularly when MRI fails to define a lesion despite high clinical suspicion [65].

Other radiotracers that have also been investigated include ^13^N-ammonia, ^18^F-fluorodeoxyglucose (FDG), fluorine-18 choline and CRH-stimulated MRI, ^18^F-FDG and ^68^Ga-DOTATATE [67].

Overall, while functional imaging is not a replacement for MRI and is not required for routine evaluation of most sellar masses, it plays a crucial role in targeted, problem-solving scenarios. These include MRI-negative Cushing’s disease, complex postoperative anatomy, discordant biochemical and anatomical findings, and selected cases where the therapeutic approach depends on confirming the functional activity of a suspected lesion.

### 3.4. Immunohistochemistry (IHC), Transcription Factors and Molecular Diagnostics—Biomarkers

In pituitary tumors, genetic alterations that influence their growth, hormone production, and invasiveness occur frequently. These alterations, affecting oncogenes or tumor suppressor genes, modulate critical signaling pathways governing cell proliferation, differentiation, and apoptosis. Significant molecular aberrations include mutations in genes such as *GNAS*, *USP8*, and *BRAF*, which are often involved in specific functional subtypes of pituitary adenomas, driving aberrant hormonal secretion and cellular proliferation [68]. Moreover, dysregulation of signaling pathways, including the MAPK/ERK, PI3K/Akt/mTOR, and Wnt/β-catenin pathways, is commonly observed, contributing to tumorigenesis and progression [69,70]. The identification of specific genetic mutations, such as those in the *PIK3CA* gene, can indicate heightened activity of downstream pathways like PI3K/Akt/mTOR, which are frequently implicated in tumor growth and resistance to conventional therapies [71]. Other notable pathways include the Gsα/protein kinase A/cAMP pathway, implicated in numerous PitNETs and syndromes like McCune-Albright, and the Sonic Hedgehog pathway, observed in various tumors including medulloblastomas [70]. Concurrently, mutations in RAS genes and PPARG/PAX8 fusions result in increased PI3K/AKT signaling, contributing to greater tumor aggressiveness [72].

The integration of pituitary hormones, lineage transcription factors, as well as cytokeratine and p53 staining into routine immunohistochemical panels, and even more specific molecular markers in selected cases, may decrease diagnostic uncertainty in rare or atypical cases where traditional histopathology is inconclusive [73,74].

Expression of anterior pituitary hormone transcription factors, such as TPIT and SF1, can differentiate “true” null-cell adenomas from gonadotroph tumors or those with aggressive biologic behavior (e.g., sparsely granulated corticotroph adenomas and Crooke cell adenomas). Additionally, TTF-1 positivity is associated with posterior pituitary lesions, including pituicytoma and granular cell tumors, and metastatic lesions from the thyroid or lungs [75]. Cytokeratin immunohistochemistry is useful for both differential diagnosis and prognostic assessment of PitNET subtypes. In GH-secreting tumors, cytokeratin expression reflects the granulation pattern (perinuclear staining in densely granulated tumors and fibrous bodies in >70% of cells in sparsely granulated tumors), whereas strong diffuse cytoplasmic staining is characteristic of corticotroph tumors [74]. Moreover, the detection of Gsα gene mutations, frequently observed in GH-secreting adenomas, elucidates the constitutive activation of the cAMP pathway and informs targeted therapeutic approaches. GNAS somatic mutations have been traditionally associated with better response to first-generation SRLs [76]. However, this has not been validated in larger series, although tumors bearing GNAS mutations tend to be of smaller size [56].

On the contrary, dysregulation within the RAF/MEK/ERK and PI3K/AKT/mTOR pathways was associated with tumor aggressiveness, as data from tissue material and cell-culture analyses suggest. Thus, measurement of the expression levels of related factors of these pathways in tissue samples of patients with PitNETs may serve as a predictor of their biological behavior [77], complementary to the Ki67 proliferation index and P53. In particular, p53 overexpression has been associated with more aggressive biological behavior, including progression of residual tumor, recurrence, and cavernous sinus invasion; however, findings across studies remain inconsistent [18,74,78].

The application of novel IHC panels may also aid in the diagnosis of other, less common sellar lesions. In some cases, the differential diagnosis of cystic sellar lesions (e.g., RCC, papillary and adamantinomatous craniopharyngiomas) can be challenging. Epithelial cell adhesion molecule (EpCAM or CD326) expression was significantly greater in cases of ACP and RCC compared to PCP and hence may constitute an alternative valuable diagnostic tool [79]. Moreover, BRAF V600E IHC may also help in differentiating PCP and RCC, particularly in cases with squamous cell metaplasia or aggressive behavior [80]. In a retrospective study of 11 surgically treated patients with RCC, the application of BRAF IHC led to the identification of one case with recurrent PCC, which had been erroneously diagnosed as RCC. Hence, BRAF IHC may be of clinical value in similar scenarios, considering also the efficacy of BRAF/MEK inhibition in PCP [80,81]. Other applications may include SSTR2 expression (meningiomas and neurocytomas), OCT4 (germinomas), TTF-1 and GATA3 (metastatic disease) and SMARCB1/INI1 (atypical teratoid tumors) [82].

The IHC profiles of sellar lesions are summarized in Table 2.

As far as molecular diagnostics are concerned, targeted sequencing and DNA methylation profiling have reemerged at the forefront of the contemporary diagnostic workup of sellar lesions. In PitNETs, integration of germline testing for established and emerging predisposition genes (including MEN1, AIP, PRKAR1A, CDKN1B, SDHx, and newer candidates such as PAM and CHEK2) has improved recognition of familial and syndromic disease, while somatic profiling has clarified subtype-specific drivers predicting the biological behavior, such as GNAS mutations in somatotroph tumors, USP8 mutations in corticotroph tumors and ESR1 mutations in prolactinomas. Although routine tumor sequencing is not yet standard for most PitNETs, expanding genomic and copy-number analyses are increasingly informative in aggressive or treatment-refractory cases and may guide enrollment in targeted or basket trials. In craniopharyngiomas, molecular diagnostics have become directly clinically actionable, with near-universal CTNNB1 and BRAF V600E mutations defining ACP and PCP, respectively. As previously mentioned, combined IHC and next-generation sequencing may now facilitate definitive diagnosis in challenging cases and selection of targeted BRAF/MEK inhibitor therapy. Detection of tumor-specific DNA methylation markers in blood (liquid biopsy) allows for non-invasive diagnosis and differentiation of PitNETs from other sellar lesions, with high accuracy (>90%), while ML-based DNA methylation classifiers now enable precise tumor classification, even in diagnostically ambiguous cases [83,84].

Collectively, these developments exemplify a bench-to-bedside paradigm in the sella, whereby molecular testing is transitioning from a purely classificatory role to a tool that informs prognosis, therapeutic stratification, and personalized clinical decision-making.

## 4. Novel Aspects in Management of Pituitary Lesions

### 4.1. Surgical Management

Surgical resection remains the cornerstone in the management of pituitary lesions, providing histopathological diagnosis, decompression of adjacent neurovascular structures, and durable disease control [85]. Over the past two decades, advances in endoscopic transsphenoidal techniques have markedly improved visualization of the sellar and parasellar regions, enabling more complete tumor resection and reduced morbidity compared with traditional transcranial approaches [86]. Endoscopic surgery is a safe and effective surgical method for a wide range of parasellar lesions, particularly when performed in high-volume, specialized centers [87]. Favorable outcomes have been reported in terms of tumor control, visual recovery, and complication rates [87]. Despite these advances, surgical management remains complex owing to the heterogeneity of pituitary lesions, variable patterns of cavernous sinus invasion, and the difficulty of balancing maximal tumor resection against preservation of endocrine and neurological function [88]. These challenges highlight the importance of multidisciplinary management and continued refinement of surgical strategies.

Advances in imaging have drastically improved preoperative planning [89]. In addition, machine learning–based prognostic models are increasingly being developed to support individualized risk stratification by capturing nonlinear relationships across multimodal datasets, including histopathological, imaging, and electronic health record notes [90].

Visualization remains an important challenge in pituitary surgery, given the depth, narrowness, and anatomical variability of the surgical corridor [91]. The transition from microscopic to endoscopic surgery has markedly improved the field of view, yet most endoscopes remain two-dimensional, requiring surgeons to infer depth through indirect cues [87]. High-definition imaging has improved tissue discrimination and may reduce tumor remnants [92]. Three-dimensional endoscopy further enhances depth perception and has demonstrated utility in complex and extended endonasal approaches, though issues such as user discomfort and workflow disruption persist [93].

Conventional surgical navigation relies on preoperative imaging and probe-based localization of vascular structures to guide the surgeon within the surgical field [94]. Although these systems have improved surgical safety, their utility is limited by workflow interruption during surgery and inaccuracy caused by intraoperative tissue shift following tumor debulking [94]. These limitations have driven the exploration of newer real-time navigation techniques [95]. Intraoperative MRI can improve identification of safely resectable tumor remnants, enhance the extent of resection, and support the identification of critical neurovascular structures [96]. However, intraoperative MRI remains resource-intensive, requires substantial operating room modification, and disrupts surgical workflow, thereby prolonging operative times [96]. Intraoperative ultrasound is a more accessible alternative to intraoperative navigation, with lower cost and reduced workflow disruption [97]. Unlike Doppler techniques focusing on vascular identification, ultrasound aims to delineate tumor tissue and reveal tumor–gland borders [97]. Advances in ultrasound probe size and image quality have addressed technical barriers, and emerging clinical studies support its feasibility and safety [98]. In addition, the integration of augmented reality (AR) in navigation platforms represents a further step toward real-time guidance. By overlaying three-dimensional anatomical models directly onto the endoscopic surgical view, AR systems eliminate the need for navigation probes and separate monitors [99].

Transsphenoidal surgery remains the treatment of choice for patients with confirmed Cushing disease [100]. MRI-negative Cushing disease represents a diagnostic and therapeutic challenge, as a substantial proportion of ACTH-secreting pituitary microadenomas are below the spatial resolution of conventional imaging [101]. Surgical management in MRI-negative cases has evolved with the adoption of the above-mentioned modern intraoperative techniques. High-definition endoscopic visualization and neuronavigation, as well as surgical strategies including selective adenomectomy or hemihypophysectomy guided by IPSS lateralization, have been increasingly used to optimize outcomes [102]. Evidence from recent clinical series and systematic reviews suggests that the use of these modern surgical approaches in experienced centers can achieve remission in a substantial proportion of patients with MRI-negative Cushing disease, supporting surgery as an effective therapeutic option despite the absence of a radiologically visible lesion [101].

Advances in optical imaging and novel tracers have improved tumor identification [103]. Fluorescent dyes, including indocyanine green, fluorescein, and folate receptor-targeted agents, have shown variable utility in distinguishing tumor tissue [103]. More advanced methods, such as probe-based confocal endomicroscopy and hyperspectral imaging, allow microstructural and biochemical analysis of tissues, facilitating differentiation between tumor and normal gland [104,105]. Furthermore, the development of exoscopes, robotic endoscope holders, and robotic endoscopes with adjustable viewing angles has improved surgical ergonomics and reduced surgeon fatigue while maintaining visualization quality [106,107].

Limited intraoperative freedom and the need for careful force control contribute to the steep learning curve of pituitary surgery [108,109]. Recent advances in surgical robotics have enabled the development of miniaturized surgical instruments with enhanced kinematic precision and haptic feedback [109]. While large telesurgical systems remain unsuitable for endonasal approaches, flexible robotic platforms show promise in preclinical settings [106]. Shared-control “smart instruments,” which augment rather than replace the surgeon’s movements, have demonstrated improved dexterity and ergonomics [106]. These instruments not only enhance surgical performance but also generate high-resolution surgical data that can be utilized for training and decision support [106]. Systematic evaluation of safety, effectiveness, and cost–benefit is essential prior to widespread adoption of these methods.

Postoperative care presents additional challenges, particularly regarding prediction of complications and long-term outcomes [110]. Emerging biomarkers, including advanced retinal imaging techniques and patient-reported outcomes collected via mobile platforms, may further enhance predictive accuracy, although careful attention to data bias and accessibility is required to ensure proper application [111,112].

These advances signal a transition toward advanced pituitary surgery, in which real-time data integration, intelligent decision support, and personalized risk stratification enhance surgical precision, training, and patient outcomes [86].

### 4.2. Medical Therapy

Medical therapy plays a central role in the management of pituitary lesions, serving as first-line treatment for selected tumor subtypes and as an adjunct to surgery or radiotherapy in cases of persistent or recurrent disease [113]. Therapeutic efficacy is largely determined by tumor phenotype, receptor expression, and downstream signaling pathways [114].

#### 4.2.1. Prolactinomas

Dopamine agonists (DAs) are the treatment of choice for prolactinomas, reflecting the high expression of dopamine receptor subtype 2 (DRD2) in lactotroph cells. Cabergoline is preferred among other DAs due to its superior efficacy, prolonged half-life, and favorable tolerability profile [115]. Most patients achieve normalization of prolactin levels and significant tumor shrinkage, including those with macro- and giant prolactinomas, frequently obviating the need for surgery [116]. Management of DA-resistant prolactinomas remains challenging, as surgical debulking and radiotherapy frequently fail to achieve prolactin normalization, while switching between dopamine agonists is generally ineffective [117]. In the absence of large clinical trials, alternative medical strategies are explored on an individualized basis [118]. While somatostatin receptor type 2 (SSTR2) is expressed in prolactinomas, somatostatin receptor ligands (SRLs) show limited efficacy, though combination therapy with dopamine agonists may improve disease control [119]. Pasireotide may be beneficial in tumors with significant SSTR5 expression, underlining the importance of molecular markers of response mentioned earlier [120]. Surprisingly, metformin has emerged as a potential adjunctive therapy owing to its antitumoral effects mediated through AMP-activated protein kinase (AMPK) activation and subsequent inhibition of mammalian target of rapamycin (mTOR) signaling [121]. Preclinical studies and limited clinical data suggest partial efficacy in dopamine agonist–resistant prolactinomas, particularly when combined with cabergoline [122]. Epidermal growth factor receptor (EGFR) signaling contributes to prolactinoma pathogenesis, and preclinical data indicate that EGFR inhibitors such as gefitinib may reduce tumor growth and prolactin secretion [123]. Additional targets include transforming growth factor (TGF)-β1 signaling and MAPK and PI3K/AKT/mTOR pathways, with mTOR inhibitors demonstrating promise in experimental models [124].

#### 4.2.2. Somatotropinomas

In acromegaly, surgery remains the preferred initial treatment when feasible [125]. Somatostatin receptor ligands (SRLs), primarily octreotide and lanreotide, represent the mainstay of medical therapy in patients with persistent disease, high surgical risk, or surgery refusal, as well as preoperatively in cases of severe disease [126]. Biochemical control and tumor shrinkage are achieved in a substantial subset of patients, with response influenced by granulation pattern, receptor density, and genetic background [127]. Pasireotide, a multireceptor SRL, offers improved efficacy in resistant disease but is frequently associated with hyperglycemia [128,129]. Dopamine agonists may be useful in mildly active disease or as adjunctive therapy [130]. Pegvisomant, a growth hormone receptor antagonist, is highly effective in normalizing IGF-1 levels; therefore, it is usually used in combination with SRLs, although it lacks direct antitumoral effects and may be associated with tumor growth [131]. The PAPE study demonstrated that switching well-controlled acromegaly patients on first-generation SRLs plus pegvisomant to pasireotide-LAR leads to maintained control of IGF-1 while substantially lowering pegvisomant doses [132]. Paltusotine, a newly approved oral nonpeptide SSTR2 agonist, demonstrates potent suppression of GH and sustained IGF-1 reduction [133].

Recent advances in the pharmacological management of acromegaly have focused on improving SRL delivery and targeting the GH axis [125]. Novel formulations of octreotide have been developed to overcome the limitations of injectable therapies, such as reduced adherence and injection burden [134]. Oral octreotide acetate demonstrated pharmacokinetic profiles comparable to subcutaneous octreotide, with sustained biochemical control in patients previously controlled on injectable SRLs [135]. Clinical trials confirmed its efficacy in maintaining IGF-1 normalization, with adverse effects consisting of mild to moderate gastrointestinal adverse events and musculoskeletal disorders [136]. A subcutaneous octreotide depot showed higher bioavailability and more rapid suppression of IGF-1 compared with long-acting release (LAR) formulations, while maintaining a favorable safety profile [137]. Nasal octreotide, including the newer DP1038 formulation with enhanced mucosal absorption, achieved GH suppression comparable to subcutaneous octreotide, supporting the feasibility of non-injectable delivery strategies. Beyond reformulations of established SRLs, several new agents targeting the GH axis are under development [138]. These include oral nonpeptide SSTR2 agonists, such as paltusotine, which demonstrated potent suppression of GH and sustained IGF-1 reduction in early-phase studies, as well as multi-receptor ligands, which showed efficacy in suppressing GH secretion, including some octreotide-resistant tumors [139,140]. Additional new orally available SSTR2 agonists and antisense oligonucleotides targeting GH receptor mRNA have entered early clinical trials, with preliminary evidence of IGF-1 reduction [141]. Dopastatins represent chimeric molecules with combined somatostatin and dopamine receptor activity designed to enhance inhibitory signaling [142]. Preclinical and clinical studies of newer dopastatins, especially TBR-065, demonstrated robust GH suppression and increased apoptotic effects in GH-secreting pituitary tumors in animal models, as well as significant decreases in GH and IGF-1 in humans, albeit with dose-dependent adverse effects, such as orthostatic hypotension [143].

#### 4.2.3. Corticotropinomas

CD is primarily managed surgically; however, medical therapy is essential in persistent, recurrent, or inoperable cases [144]. Pituitary-directed therapies include pasireotide and cabergoline, targeting SSTR5 and DRD2, respectively [145,146]. Pasireotide achieves cortisol normalization in a subset of patients and may induce tumor shrinkage but frequently impairs glucose metabolism [145]. Cabergoline demonstrates variable and often transient efficacy [146]. Adrenal-directed steroidogenesis inhibitors—such as ketoconazole, metyrapone, and osilodrostat—constitute major therapeutic options, while glucocorticoid receptor antagonist mifepristone improves clinical features but precludes biochemical monitoring due to its mechanism of action [144].

Increasing insight into the molecular pathogenesis of CD has led to the development of additional therapeutic agents [144]. Retinoic acid has demonstrated inhibitory effects on ACTH secretion, cortisol production, and corticotroph cell proliferation, while also modulating adrenal steroidogenesis [110,147]. Prospective clinical studies in patients with persistent or recurrent CD demonstrated significant and sustained reductions in urinary free cortisol (UFC), although disease relapse was observed in some cases [147]. Abnormal activation of the EGFR signaling pathway, frequently associated with somatic ubiquitin-specific protease 8 (USP8) mutations, has been implicated in corticotroph tumorigenesis [148]. Preclinical studies have demonstrated that inhibition of EGFR signaling and its modulators can suppress ACTH secretion and tumor growth [149]. Dysregulated glucocorticoid receptor signaling due to overexpression of heat shock protein (HSP90) has emerged as a key mechanism of glucocorticoid resistance in corticotroph adenomas, with HSP90 inhibition restoring cortisol levels and demonstrating anti-proliferative effects [150]. Additional investigational strategies include targeting BRAFV600E mutations in USP8-wild-type tumors, inhibition of cyclin-dependent kinases (R-Roscovitine) involved in cell-cycle regulation, and modulation of other receptors and transcriptional regulators implicated in ACTH synthesis and corticotroph proliferation, such as arginine-vasopressin receptor and nuclear testicular orphan receptor 4 [151,152,153,154]. Blockade of glucocorticoid receptor signaling with selective antagonists such as relacorilant represents a complementary therapeutic approach currently under clinical evaluation [155].

#### 4.2.4. Thyrotropinomas and Non-Functioning Pituitary Tumors

TSH-secreting tumors are rare and are preferentially treated surgically [156]. SRLs effectively suppress hormone secretion and may induce tumor shrinkage in most patients, whereas dopamine agonists show inconsistent efficacy [156]. While no novel medical therapies are currently under active investigation for TSH-secreting pituitary adenomas, isolated clinical observations suggest that somatostatin multireceptor ligands may achieve biochemical control in selected cases [157].

Non-functioning pituitary lesions are primarily managed surgically, with radiotherapy reserved for progressive disease [158]. Dopamine agonists may induce tumor stabilization or shrinkage in selected cases, whereas SRLs have limited efficacy [159]. Medical treatment options for non-functioning pituitary tumors (NFPTs) remain limited, despite the high prevalence of this tumor subtype among newly diagnosed pituitary neoplasms [160]. NFPTs of gonadotroph origin typically exhibit a predominance of SSTR3 expression. Preclinical studies have demonstrated that SSTR3 agonists could be an important agent for residual or recurrent NFPT refractory to surgery or radiotherapy [161]. Additional in vitro data indicate that dopastatins can also reduce NFPT cell viability, although the efficacy of newer compounds remains to be established [142]. Furthermore, emerging evidence shows that immunomodulatory strategies targeting macrophages or T lymphocytes may represent future therapeutic targets [162].

#### 4.2.5. Other Sellar Lesions

Current treatment of craniopharyngiomas mainly consists of partial resection and adjuvant radiotherapy due to the high morbidity of aggressive total resection. Intracystic treatments such as bleomycin or interferon-α have also been used to control cystic fluid formation and reduce cyst size. Despite these options, traditional local therapies are far from ideal due to significant complications and negative impact on quality of life. Recent advances in understanding craniopharyngioma biology have enabled targeted molecular therapies that exploit distinct genetic drivers in the two major subtypes [163]. Adamantinomatous craniopharyngiomas often harbor β-catenin and MAPK/ERK pathway abnormalities [164,165]. Agents such as MEK inhibitors (e.g., binimetinib) and immunotherapy agents (e.g., tocilizumab, interferon) have been administered with mostly favorable responses in case reports and small series [166,167]. Papillary craniopharyngiomas, which harbor BRAF-V600E mutations, have responded particularly well to BRAF inhibitors (dabrafenib, vemurafenib), either alone or combined with MEK inhibitors (trametinib, cobimetinib) [168,169,170].

Emerging medical treatments for meningiomas are increasingly focused on molecularly targeted strategies that move beyond traditional cytotoxic chemotherapy and hormonal agents, which historically have shown limited benefit [171]. Novel therapies under investigation include inhibitors targeting key oncogenic pathways such as focal adhesion kinase, sonic hedgehog, phosphoinositide-3-kinase (PI3K), and cyclin-dependent kinases, tailored to tumors with specific genetic alterations [81,172]. Additionally, meningiomas ubiquitously express SSTR2, prompting active exploration of therapeutic approaches such as peptide receptor radionuclide therapy (PRRT) [173].

The primary medical treatment strategy for intracranial germinoma centers on systemic chemotherapy, particularly in combination with reduced-dose radiation to minimize long-term toxicity. Molecularly targeted therapy is also increasingly integrated into neuro-oncology [174]. Whole-exome sequencing studies have demonstrated recurrent alterations in the KIT and KRAS signaling pathways, providing a biologic rationale for targeted inhibition [175]. Dasatinib, a multi-tyrosine kinase inhibitor with activity against KIT and theoretical blood–brain barrier penetration, has shown feasibility in early pilot studies involving patients with newly diagnosed and recurrent germinoma [176]. Medical treatment of primary pituitary lymphoma is based on systemic chemotherapy regimens used for primary central nervous system lymphoma (PCNSL), with high-dose methotrexate combined with other agents, most notably rituximab, as the mainstay of medical treatment [177]. Emerging immune-based and targeted therapies, including immune checkpoint inhibitors, chimeric antigen receptor T cell therapy, and Bruton tyrosine kinase inhibition with ibrutinib, have demonstrated promising activity in relapsed/refractory PCNSL. Molecular insights into B-cell receptors and PI3K/mTOR signaling may lead to the development of newer therapeutic targets. These advances may also have translational relevance for pituitary lymphoma sharing similar biological drivers [178].

The management of hypophysitis is guided by disease severity, mass effect, and the presence of pituitary hormone deficiencies. High-dose glucocorticoids (GCs) represent the cornerstone of therapy, targeting the underlying inflammatory process and inducing both radiologic and partial hormonal recovery. For patients with mild or asymptomatic disease, clinical observation with periodic MRI and endocrine reassessment may be appropriate, as spontaneous improvement may occur. In GC-refractory or relapsing cases, immunosuppressive agents such as azathioprine, methotrexate, mycophenolate mofetil, or rituximab can be employed as alternatives, particularly in B-cell predominant or IgG4-related disease. Surgical intervention is reserved for severe mass effect, optic chiasm compression, or diagnostic uncertainty, whereas radiotherapy, including fractionated or stereotactic modalities, may be considered in treatment-resistant or recurrent cases. Hormone replacement therapy remains important throughout management, ensuring stabilization of endocrine function while the underlying inflammatory process is controlled [179].

Overall, medical therapies for pituitary lesions are highly effective when tailored to tumor subtype and molecular characteristics, supporting a personalized approach to long-term disease control.

### 4.3. Radiotherapy

Radiotherapy is an established component of multidisciplinary management of pituitary lesions in selected cases of residual, recurrent, or progressive disease following surgical resection as a third-line therapy [180]. Conventionally fractionated external beam radiotherapy (EBRT) remains the most widely utilized and extensively studied method [181]. Advances in EBRT delivery, including three-dimensional conformal radiotherapy (CRT), intensity-modulated radiotherapy (IMRT), and volumetric modulated arc therapy (VMAT), have improved targeting of and reduced irradiation of surrounding critical structures such as the optic apparatus and temporal lobes. Despite these improvements, radiation-induced hypopituitarism remains the most common late adverse effect [180]. Visual complications are uncommon, and neurocognitive deficits are generally mild, while cerebrovascular events and secondary intracranial neoplasms have also been reported, particularly with extended follow-up [182]. Hypofractionated stereotactic radiotherapy is a recent advance in EBRT, demonstrating favorable short-term control and acceptable toxicity profiles, although long-term outcomes remain incompletely defined [183].

Stereotactic radiosurgery (SRS) is a highly focused method of radiotherapy that delivers a single, high-dose fraction of ionizing radiation, allowing precise targeting of pituitary lesions while minimizing radiation exposure to adjacent critical structures [184]. SRS can be delivered using several platforms, including linear accelerator–based systems, CyberKnife, proton beam radiosurgery, and Zap-X, all of which are designed to achieve highly conformal dose distributions [185,186,187]. SRS is most commonly utilized as an adjuvant or salvage therapy following surgical resection, although it can also be utilized as a primary treatment in selected patients who are not surgical candidates or who decline surgery [184].

Emerging radiotherapy modalities aim to further improve dose precision and reduce treatment-related toxicity [180]. Proton beam therapy utilizes the physical properties of protons to deposit radiation energy directly at the tumor depth, minimizing dose to surrounding normal tissues [188]. Clinical series have reported tumor control rates of approximately 98% at a median follow-up of 3.5 years; however, the clinical adoption of proton therapy is currently limited by high cost, restricted availability, and a lack of long-term comparative outcome data [189]. PRRT represents a novel, targeted radiotherapeutic strategy for aggressive or refractory pituitary tumors expressing somatostatin receptors. This approach utilizes radiolabeled peptides, most commonly with Lutetium-177, to deliver intracellular radiation following receptor-mediated internalization [190]. Clinical responses to PRRT have been heterogeneous, with partial tumor reduction observed in some patients and minimal response in others [191,192]. Reported adverse effects include cytopenias, facial pain, and rare cases of pituitary apoplexy [193]. At present, PRRT remains investigational, with its broader application limited by the absence of standardized dosing protocols and insufficient long-term efficacy data [190]. These emerging modalities highlight an evolving radiotherapeutic landscape in pituitary lesion management, with ongoing efforts focused on optimizing precision, minimizing toxicity, and defining appropriate patient selection.

## 5. Conclusions

The diagnosis and management of sellar lesions is becoming increasingly intricate, demanding a sophisticated approach that synthesizes conventional histopathological findings with cutting-edge molecular insights. Sellar lesions represent a broad spectrum of neoplastic and non-neoplastic conditions, making precise differential diagnosis challenging due to overlapping clinical, radiological, and endocrinological presentations. PitNETs, while the most common, must be carefully distinguished from other entities such as RCCs, meningiomas, craniopharyngiomas, and even rare metastatic lesions, each requiring distinct therapeutic strategies.

Advancements in molecular biology have significantly elucidated the mechanisms underlying pituitary tumorigenesis and progression. Specific genetic alterations are frequently associated with particular PitNET subtypes, leading to abnormal hormone secretion and increased cellular proliferation. Moreover, dysregulation of key signaling pathways is a common feature, contributing to tumor initiation and growth. Management of sellar lesions requires a multidisciplinary approach that integrates clinical, radiological, endocrinological, and pathological findings to ensure accurate diagnosis and optimal treatment. Individualized management strategies are essential, as outcomes depend on lesion type, extent, hormonal activity, and the presence of local invasion or complications.

## Figures and Tables

**Table 1:** Sellar lesion categories.

Category	Subcategory	Tumor Type	Key Diagnostic Features
**Tumors**	PitNET	Lactotroph Adenoma	Most common type; secretes prolactin. Immunopositivity for prolactin.
		Somatotroph Adenoma	Secretes growth hormone. Can cause acromegaly. Immunopositivity for GH.
		Corticotroph Adenoma	Secretes ACTH. Causes Cushing’s disease. Immunopositivity for ACTH.
		Thyrotroph Adenoma	Secretes TSH. Rare. Immunopositivity for TSH.
		Gonadotroph Adenoma	Secretes FSH and LH (often one or both). Frequently non-functioning. Immunopositivity for FSH and/or LH.
		Null Cell Adenoma	Non-functioning. No hormone secretion. Negative for pituitary hormones by immunohistochemistry.
		Plurihormonal Adenoma	Secretes multiple hormones. Requires careful immunohistochemical analysis.
	High-grade Pituitary Tumors	Atypical Pituitary Adenoma	Increased mitotic activity, p53 immunoreactivity, Ki-67 labeling index > 3%.
		Pituitary Carcinoma/Metastases	Rare. Defined by the presence of distant metastasis.
	Other Sellar Region Tumors	Craniopharyngioma	Adamantinomatous and papillary subtypes. Often cystic. Arise from remnants of Rathke’s pouch.
		Rathke Cleft Cyst	Benign cyst lined by ciliated epithelium. Located in the pituitary gland or suprasellar region.
		Pituicytoma/Spindle cell oncocytoma	Rare tumors of the posterior pituitary.
		Granular Cell Tumor	Benign tumor with granular cytoplasm. S-100 positive. Heterogeneous and hyperdense on CT.
		Pituitary lymphoma	Rare. Vivid heterogeneous enhancement.
**Cellular Infiltrates**	Histiocytosis	Langerhans cell histiocytosis	Most common form.
		Erdheim–Chester disease	Rare infiltrative disorder.
	Hypophysitis	Granulomatous	e.g., neurosarcoidosis, tuberculosis.
		Lymphocytic	Autoimmune origin.
		IgG4-related	Associated with systemic IgG4-related disease.
		Xanthomatous	Rare, lipid-laden histiocytes.
**Germ cell tumors**		Germinoma	large clear cells, lymphocytes, and diffuse OCT4/SALL4/KIT positivity
		Non-germinomatous cell tumors	Aggressive, heterogeneous tumors (e.g., yolk sac, choriocarcinoma, embryonal, mixed) characterized by elevated a-FP and/or β-HCG,
Other Lesions	—	Pituitary abscess	Peripheral enhancing cystic lesion. Diffusion restriction.
		Pituitary stone	Low signal lesion. Enlarged sella turcica

Abbreviations: ACTH, adrenocorticotropic hormone; a-FP: Alpha-fetoprotein; β-HCG: beta human chorionic gonadotropin; CT, computed tomography; FSH, follicle-stimulating hormone; GH, growth hormone; LH, luteinizing hormone; OCT4: Octamer-binding transcription factor 4; PitNET, pituitary neuroendocrine tumor; SALL4: Sal-like protein 4; TSH, thyroid-stimulating hormone.

**Table 2:** Immunohistochemical profile and characteristic staining patterns of sellar lesions.

Lesion	Immunohistochemical Markers (Positive)	Pattern of Positivity
***Pituitary neuroendocrine tumors (PitNETs)***		
Somatotroph PitNET, densely granulated	PIT1, GH, CK8/18	PIT1: diffuse nuclear; GH: strong diffuse cytoplasmic; CK8/18: diffuse cytoplasmic
Somatotroph PitNET, sparsely granulated	PIT1, GH (often weak), CK8/18	PIT1: diffuse nuclear; GH: focal or weak; CK8/18: perinuclear dot-like fibrous bodies
Lactotroph PitNET, densely granulated	PIT1, PRL, ERα	PIT1: diffuse nuclear; PRL: diffuse cytoplasmic; ERα: nuclear
Lactotroph PitNET, sparsely granulated	PIT1, PRL, ERα	PRL: perinuclear/Golgi-type cytoplasmic; ERα: nuclear
Mammosomatotroph PitNET	PIT1, GH, PRL, ERα	GH and PRL: diffuse cytoplasmic; ERα: nuclear
Thyrotroph PitNET	PIT1, TSH	PIT1: diffuse nuclear; TSH: cytoplasmic, often focal
Gonadotroph PitNET	SF1, FSH, LH, α-subunit	SF1: diffuse nuclear; FSH/LH: focal cytoplasmic
Corticotroph PitNET, densely granulated	TPIT, ACTH	TPIT: diffuse nuclear; ACTH: diffuse cytoplasmic
Corticotroph PitNET, sparsely granulated/silent	TPIT	TPIT: focal or weak nuclear; ACTH absent or focal
Crooke cell tumor	TPIT, ACTH, CK8/18	ACTH: peripheral cytoplasmic; CK8/18: dense perinuclear hyaline rings
Plurihormonal PIT1-lineage PitNET	PIT1, ≥2 PIT1-lineage hormones	PIT1: diffuse nuclear; hormones variably expressed
Null-cell PitNET	—	Negative for pituitary hormones and lineage transcription factors
***Posterior pituitary tumors***		
Pituicytoma	TTF1, S100	TTF1: diffuse nuclear; S100: cytoplasmic
Granular cell tumor	TTF1, S100, CD68	TTF1: nuclear; CD68: coarse granular cytoplasmic
Spindle cell oncocytoma	TTF1, EMA, S100	TTF1: nuclear; EMA: membranous/cytoplasmic
***Craniopharyngiomas***		
Adamantinomatous craniopharyngioma	β-catenin, CK5/6	β-catenin: nuclear accumulation in whorl cells; CK5/6: epithelial
Papillary craniopharyngioma	BRAF V600E, CK7	BRAF V600E: diffuse cytoplasmic epithelial staining
***Other epithelial/cystic lesions***		
Rathke cleft cyst	CK7, EMA	CK7: luminal epithelial; EMA: luminal/membranous
Epidermoid cyst	CK5/6, p63	Diffuse squamous epithelial staining
Dermoid cyst	CK5/6	Squamous epithelium with adnexal structures
***Meningeal/mesenchymal lesions***		
Meningioma (sellar/parasellar)	EMA, PR, SSTR2A	EMA: membranous; PR: nuclear; SSTR2A: strong membranous
***Solitary fibrous tumor***	STAT6, CD34	STAT6: nuclear; CD34: diffuse cytoplasmic
***Bone/notochordal lesions***		
Chordoma	Brachyury, CK, EMA	Brachyury: nuclear; CK/EMA: cytoplasmic
Chondrosarcoma	S100	Diffuse nuclear and cytoplasmic
***Germ cell tumors***		
Germinoma	OCT3/4, PLAP, c-KIT	OCT3/4: nuclear; c-KIT: membranous
Mature teratoma Immature teratoma Yolk sac tumor Choriocarcinoma Embryonal carcinoma	No common tumor markers PLAP in mixed tumors β-HCG, AFP, SALL4, c-KIT AFP, PLAP, SALL4, c-KIT β-HCG, PLAP AFP, PLAP OCT4, CD30, SALL4, c KIT	β-HCG: focal, AFP: focal, SALL4: diffuse nuclear, c-KIT: variable Diffuse positivity β-HCG: diffuse, PLAP variable CD30: membranous, C-KIT: non-membranous
***Inflammatory/infiltrative lesions***		
Lymphocytic hypophysitis	CD3, CD20	Mixed polyclonal lymphoid infiltrate
IgG4-related hypophysitis	IgG4, IgG	Increased IgG4+/IgG+ plasma cell ratio
***Metastatic tumors***		
Metastatic carcinoma (site dependent)	Cytokeratins, organ-specific markers	Pattern dependent on primary tumor

Abbreviations: ACTH, adrenocorticotrophic hormone; AFP: alpha fetoprotein, β-HCG: beta human chorionic gonadotropin; CK, cytokeratin; EMA, epithelial membrane antigen; ERα, estrogen receptor alpha; GH, growth hormone; PitNET, pituitary neuroendocrine tumor; PLAP, placental-like alkaline phosphatase; PR, progesterone receptor; SALL4 Sal-like protein 4.

## Data Availability

No new data were created or analyzed in this study. Data sharing is not applicable to this article.

## Abbreviations

The following abbreviations are used in this manuscript:

3D-GRE	Volumetric gradient echo
ACTH	Adrenocorticotropic hormone
AI	Artificial intelligence
D2R	Dopamine-2 receptor
DMRI	Dynamic enhanced MRI
DWI	Diffusion weighted imaging
EGFR	Epidermal growth factor
FLAIR	Fluid attenuated inversion recovery
FSE	Fast spin echo
GH	Growth hormone
IPSS	Inferior petrosal sinus sampling
ML	Machine learning
NFPA	Non-functioning pituitary adenoma
PitNET	Pituitary neuroendocrine tumor
RCC	Rathke’s cleft cyst
SRLs	Somatostatin receptor ligands analogs
TGF	Transforming growth factor
